# The identification of blood pressure variation with hypovolemia based on the volume compensation method

**DOI:** 10.3389/fphys.2023.1180631

**Published:** 2023-07-27

**Authors:** Ruijuan Chen, Ming He, Shumian Xiao, Cong Wang, Huiquan Wang, Jiameng Xu, Jun Zhang, Guang Zhang

**Affiliations:** ^1^ School of Life Sciences, TianGong University, Tianjin, China; ^2^ Tianjin Key Laboratory of Quality Control and Evaluation Technology for Medical Devices, Tianjin, China; ^3^ Systems Engineering Institute, Academy of Military Sciences, People’s Liberation Army, Tianjin, China

**Keywords:** hypovolemia, volume compensation method, blood pressure variation, photoplethysmography, non-invasive

## Abstract

**Objective:** The purpose of this study is to identify the blood pressure variation, which is important in continuous blood pressure monitoring, especially in the case of low blood volume, which is critical for survival.

**Methods:** A pilot study was conducted to identify blood pressure variation with hypovolemia using five Landrace pigs. New multi-dimensional morphological features of Photoplethysmography (PPG) were proposed based on experimental study of hemorrhagic shock in pigs, which were strongly correlated with blood pressure changes. Five machine learning methods were compared to develop the blood pressure variation identification model.

**Results:** Compared with the traditional blood pressure variation identification model with single characteristic based on single period area of PPG, the identification accuracy of mean blood pressure variation based on the proposed multi-feature random forest model in this paper was up to 90%, which was 17% higher than that of the traditional blood pressure variation identification model.

**Conclusion:** By the proposed multi-dimensional features and the identification method, it is more accurate to detect the rapid variation in blood pressure and to adopt corresponding measures.

**Significance:** Rapid and accurate identification of blood pressure variation under low blood volume ultimately has the potential to effectively avoid complications caused by abnormal blood pressure in patients with clinical bleeding trauma.

## 1 Introduction

Hemorrhagic shock is a pathophysiological process characterized by reduced effective circulating blood volume and cardiac output, insufficient tissue perfusion, disordered cell metabolism and impaired function due to massive blood loss caused by trauma (Liu et al., 2015). Hemorrhagic shock is often accompanied by concomitant hypotension, which is defined as systolic blood pressure less than 90 mmHg and differential pulse pressure less than 20 mmHg (Chou et al., 2016; Tran et al., 2018). According to World Health Organization (WHO) statistics, about 10% of global deaths and 16% of disability cases are caused by trauma, which is also the leading cause of death for people under the age of 40 worldwide (World Health Organization, 2019). In trauma patients, the death rate due to excessive blood loss is about 30–40 percent (Edgard et al., 2015; Keane, 2016; Palmer, 2017), and the death rate due to incorrect treatment and inappropriate treatment is 10–20 percent (Küchler et al., 2020).

Arterial blood pressure is critical for adequate tissue perfusion, providing oxygen delivery for energy needs. Continuous and reliable measurements of absolute blood pressure are required for critically ill patients in the ICU, and variations in blood pressure of even a few minutes in patients with hypovolemic blood loss pose unpredictable risks, including hemorrhagic shock (Ameloot et al., 2013). Continuous detection of the blood pressure trend of patients in the state of blood loss and hypovolemia can provide important cardiovascular state supporting data and provide early intervention for corresponding treatment methods. The results of various studies of continuous noninvasive blood pressure monitoring devices versus invasive blood pressure monitoring methods were summarized by Kim et al. They found a significant difference between the non-invasive and invasive blood pressure obtained with the CNAP and ClearSight devices based on the volumetric compensation method. The standard deviations were 5.5 ± 9.3 mmHg and 3.5 ± 6.8 mmHg for CNAP and ClearSight respectively (Kim et al., 2014; Vos et al., 2014; Meidert and Saugel, 2017). This analysis shows that the accuracy and precision of continuous noninvasive devices are not interchangeable with invasive blood pressure measurements.

A method for blood pressure variation identification under hypovolemia based on the volume compensation method and pulse wave morphological characteristics is proposed in this study. Currently, the volume compensation method is a relatively mature blood pressure monitoring technology. This method keeps the blood volume constant in the vessel by applying a pressure value equivalent to the intravascular pressure outside the measurement (ZhangLiuChen and Liu, 2020). Studies have shown that the PPG signal profile of the photoelectric pulse wave signal is mainly controlled by the blood pressure waveform, and contains cardiovascular information, such as blood vessel stiffness and blood pressure. A large number of studies have verified that a large amount of cardiovascular information is contained in the PPG signal, which is strongly correlated to blood pressure (Mukherjee et al., 2018). The morphological analysis of PPG has been applied to vascular assessment (Fedotov, 2019a), providing rich information for cardiovascular analysis (Fedotov, 2019b; Subashini et al., 2021). There were also some studies that use morphological characteristics of not only PPG but also ECG (Electrocardiogram) signals to jointly estimate blood pressure, and to estimate SBP value every 30 s (Sun et al., 2016; Sun et al., 2022). A study that predicted blood pressure by combining various morphologies of Pulse Transit Time and PPG verified that the morphological features of PPG improved the accuracy of blood pressure estimation (Ding and Zhang, 2015; Ding et al., 2016; Lin et al., 2017; Rastegar et al., 2019).

Based on the data set under the experimental model of animal controlled hypovolemia, this study uses the photoelectric pulse signal collected by the volume compensation method to identify the variation of 11-degree blood pressure in the range of 5–15 mmHg. Five models, namely, LightGBM, Random Forest, XGBoost, CatBoost, and Decision Tree, were employed to investigate the advantages of multi-dimensional features compared with single-dimensional features in the identification of blood pressure variation under low blood volume. The accurate prediction of blood pressure variation was realized, which verifies the validity of this research method. Using accurate results of non-invasive blood pressure variation identification under hypovolemia during blood loss can not only help avoid adverse events caused by invasive blood pressure monitoring (Suess and Pinsky, 2015; Minokadeh and Pinsky, 2016), but also provide accurate diagnostic prediction for patients under cardiovascular monitoring to reduce patient tissue hypoxia, mitigate oxidative damage, prevent multiple organ failure, and improve clinical outcomes (Janssen et al., 2017; Nachman et al., 2020).

## 2 Materials and methods

### 2.1 Experiment

**FIGURE 1:**
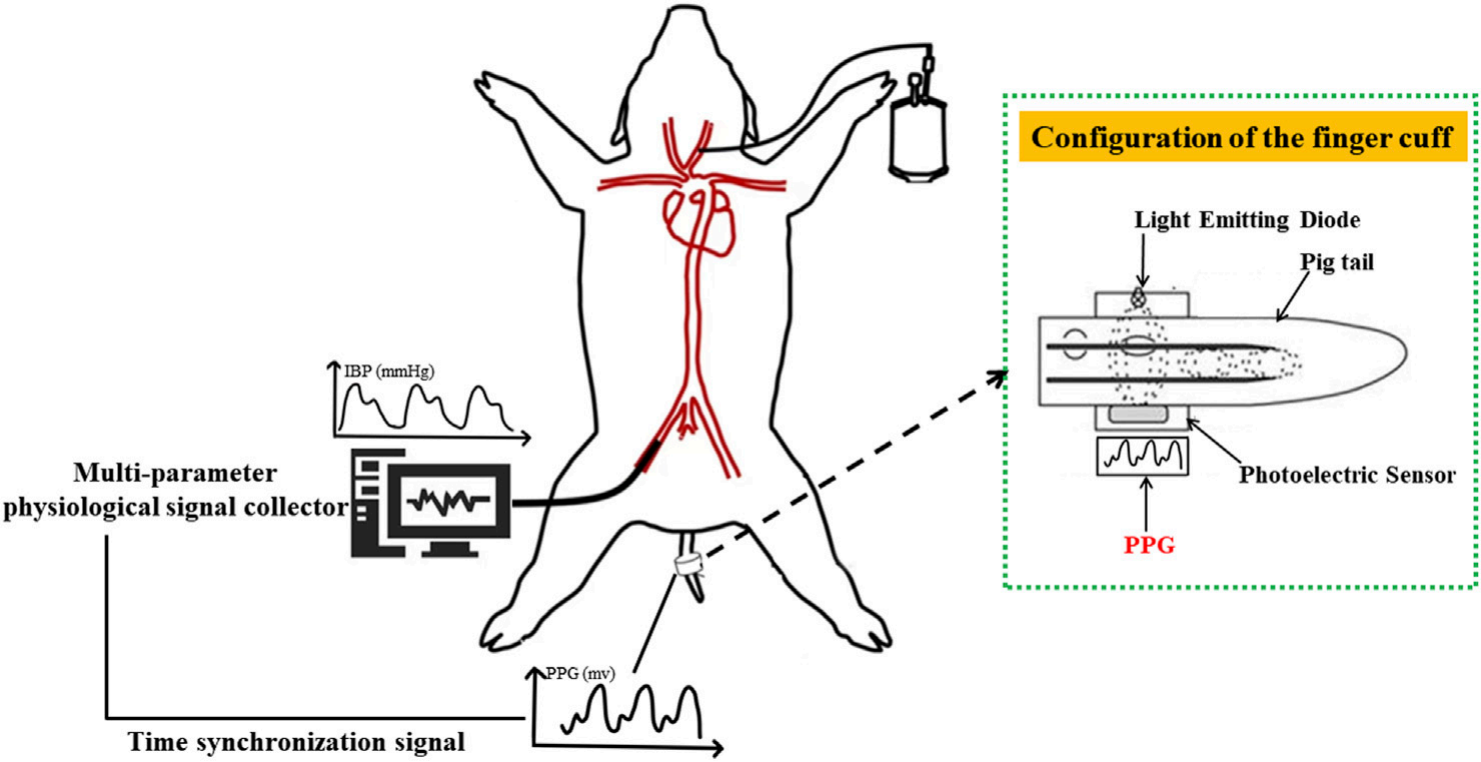
Schematic diagram of animal blood loss experiment. PPG signal (human finger probe used) under constant volume and invasive blood pressure were collected based on synchronization level signal sent by the finger cuff to achieve strict time alignment.

An animal model of hemorrhagic hypovolemia was designed in this study (The experimental schematic diagram is shown in Figure 1), and five healthy Landrace pigs weighing 23 ± 6 kg were selected as the subjects for a pilot study on blood pressure discrimination with hypovolemia. After Landrace pigs were anesthetized, the pigs were intubated and mechanically ventilated using a ventilator to prevent spontaneous breathing from affecting signal acquisition. Mindray monitor was used to monitor the physiological state and tail PI (Perfusion Index) of pigs in real time. The femoral artery was punctured on the pig, and the IBP signal was collected using the Chengdu Instrument RM6240C multi-channel physiological parameter acquisition device, while the self-developed device and deflatable optoelectronic finger cuff was used to collect the PPG signal on the pig’s tail (The light Emitting Diode inside the finger cuff emits infrared light, which is transmitted through the tissues of the pig’s tail and the arterial veins, and is received by the Photoelectric Sensor. Due to the flow of blood in the arteries, there is a change in the absorption of the light so that the transmitted light is converted into an electrical signal to form a PPG signal. So the measured PPG signal is opposite to the actual PPG signal waveform of the pig.). The sampling frequency of Chengdu Instrument RM6240C multi-channel physiological parameter acquisition instrument equipment is 1,000 Hz, and the sampling frequency of self-developed device is 500 Hz. Multiple bloodletting operations were performed through the carotid artery until the tail PI (Perfusion Index) was under 0.3, which indicated a state of hypovolemia. which indicates a state of hypovolemia. IBP and PPG signals were collected synchronously during bloodletting. The animal Invasive blood pressure span changed during the blood loss process, where the blood pressure of each animal decreased from different initial baseline blood pressure to blood pressure under hypovolemia. Experiment in this study was approved by the Medical Ethics Committee of Chinese PLA General Hospital (No. S2020-045-01).

### 2.2 Pressure setting and signal acquisition

#### 2.2.1 Constant pressure setting algorithm

**FIGURE 2:**
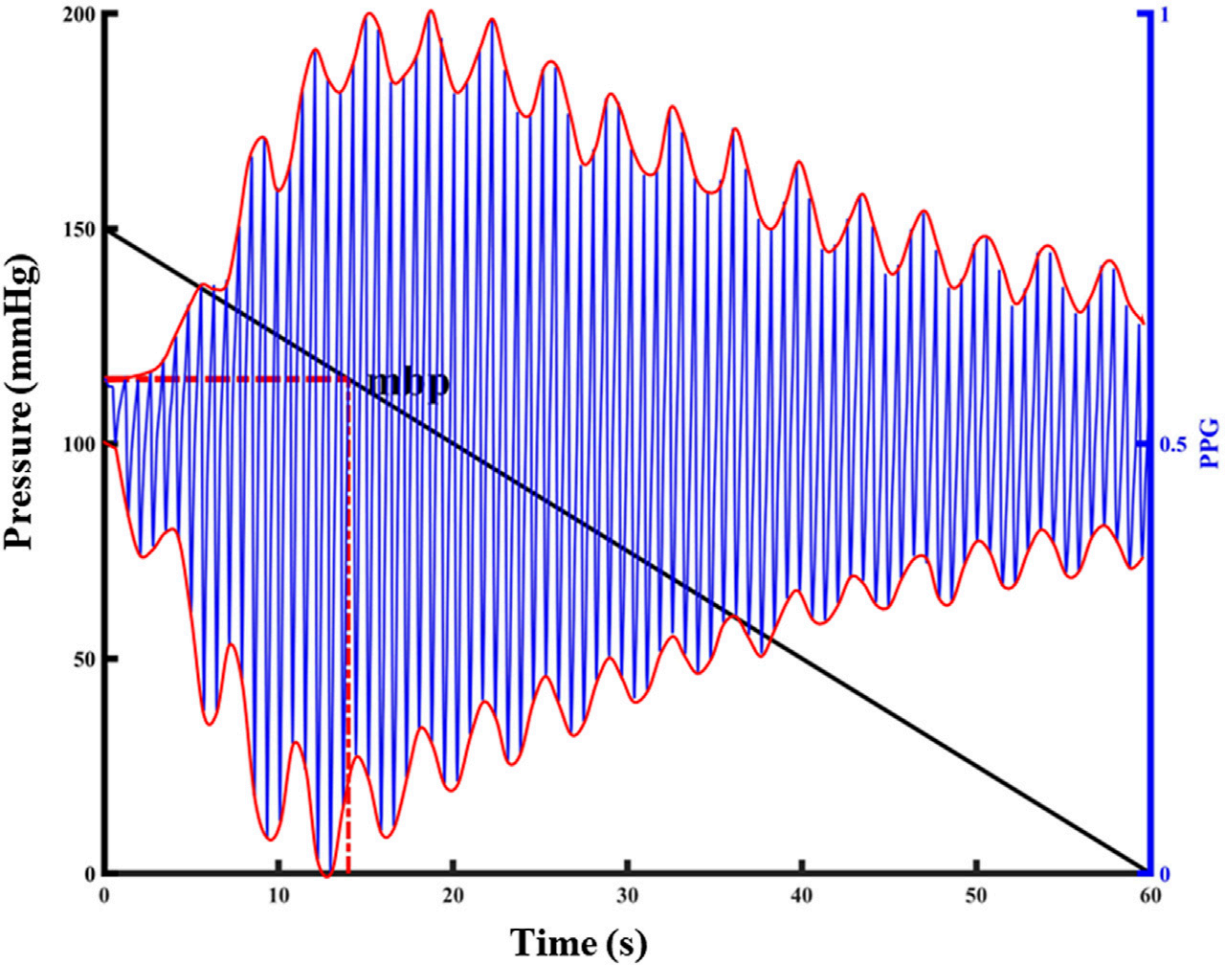
Relationship between PPG pulsation and external pressure at the detection site. The left y-label represents the externally applied pressure value, the black diagonal line in the figure represents the pressure change, the right y-label represents the normalized PPG value, the blue line in the figure represents the PPG waveform, and the red curve represents the upper and lower envelopes of the PPG waveform.

The blood pressure change identification is based on the photoelectric pulse wave signal of the detection site under constant pressure. In the process of external force change, when the external force applied to the detection site is equal to the average pressure in the artery, pulse wave peak reaches the maximum intensity. As intra-arterial blood pressure changes, the shape of the photoplethysmography wave changes significantly at the constant pressure (Figure 2).

The peak-to-peak value was calculated according to the upper and lower envelopes of the photoelectric pulse wave signal under pressure (Find the function of the PPG signal envelope and the parameters to be set: the function is *envelope*; parameter 1 is set to *250*; parameter 2 is set to “*peak*”.). The maximum peak-to-peak value corresponding to the pressure applied to the detection site was the average pressure corresponding to the detection site, and the constant pressure was set at the detection. Repeated application of 150 mmHg finger cuff pressure to the animal tail before the experiment can result in the temporary blood flow blocking effect, in addition to not causing venous congestion damage to the tail. The external force of 150 mmHg was repeatedly applied on the tail, released to 0 mmHg, and the tail photoelectric pulse wave was collected in real time. In this experiment, blood loss and transfusion were performed to change the blood pressure state continuously, and constant pressure value was calculated under a new blood pressure homeostasis. A total of 214 groups of constant pressure sample data were collected, with blood pressure ranging from 52 mmHg to 119 mmHg.

#### 2.2.2 Photoelectric pulse wave data set at constant pressure

Before each blood loss operation in the animal experiment, the constant pressure value that should be applied to the tail of the animal was calculated, and the finger cuff was inflated to this constant pressure value. On a constant pressure, the pressure was maintained for 10 min during a single bloodletting of 200 mL, the pressure was released to zero after 10 min, and the tail was relieved for 10 min. Two photoelectric pulse wave data segments of blood loss process were collected for each animal, and a total of ten blood loss data segments were collected, based on the photoelectric acquisition terminal in the tail finger cuff.

### 2.3 Data preprocessing

**FIGURE 3:**
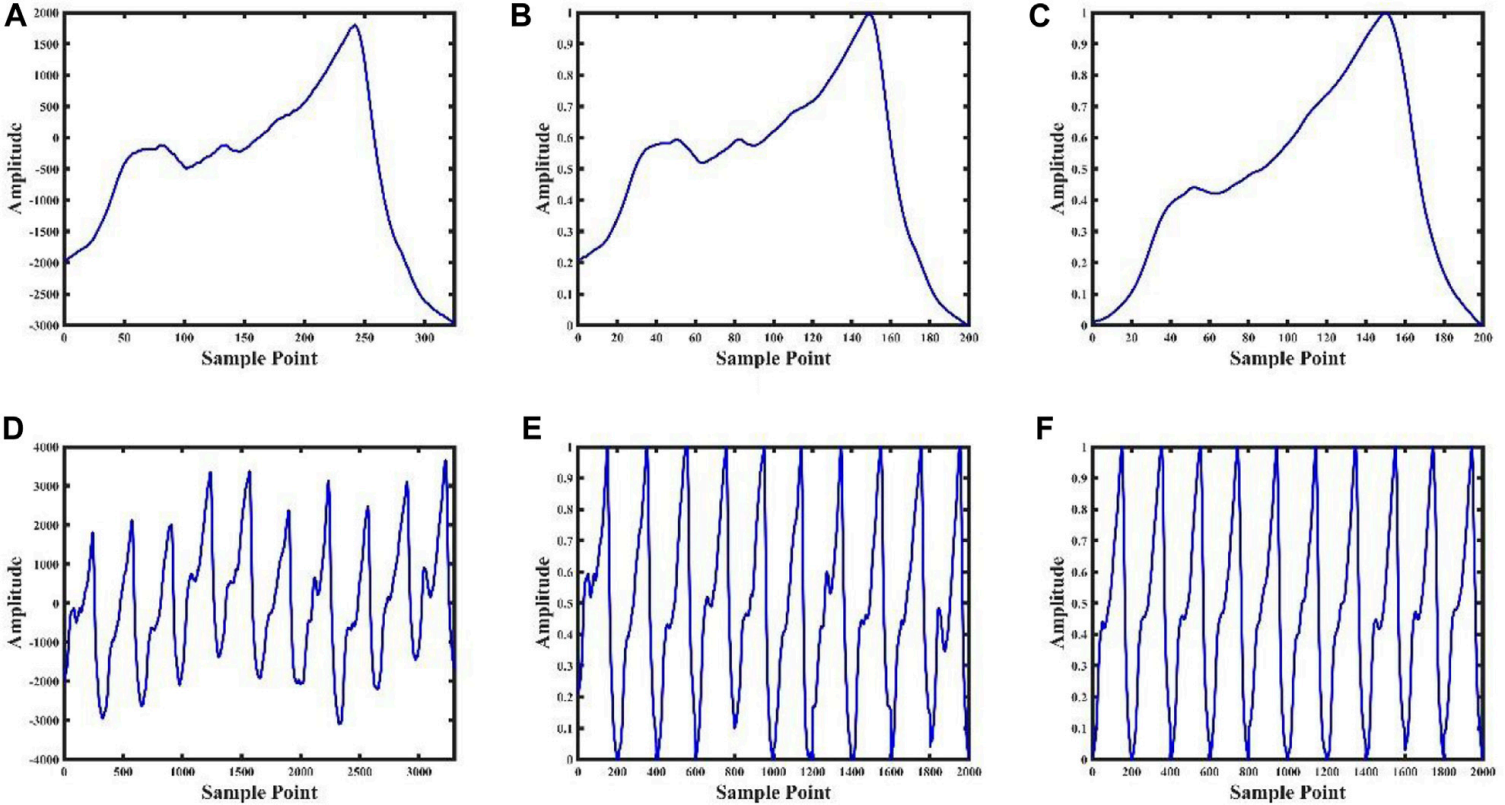
Data processing result graph. Figures **(A)**, **(B)**, **(C)** are the pulse wave waveform after single-cycle preprocessing, the pulse wave waveform after single-cycle normalization, and the pulse wave waveform after single-cycle template, respectively; Figures **(D)**, **(E)** and **(F)** are the ten consecutive cycles pulse waveforms after of preprocessing, ten consecutive cycles pulse waveforms after normalizing, and ten consecutive pulse cycles waveforms after templating, respectively.

The photoelectric pulse wave signal was collected under constant pressure in the state of continuous blood loss. The experimental subject was accompanied by an accelerated heartbeat and changes in the elasticity of blood vessels during bloodletting. To minimize the influence of factors other than intravascular pressure on the photoelectric pulse waveform, the template processing (Figure 3) for the photoelectric pulse wave was adopted. The templating process consists of five parts:

(1) Preprocessing: Check the original data and remove invalid data segments during convulsions of animals or abnormal device connections. Retain data from 0.3–20 Hz with Butterworth filter to remove baseline drift, low frequency noise and high frequency noise.
(2) Pulse wave single-cycle amplitude normalization: A pulse wave cycle from the pulse wave trough value to the next pulse wave trough value was defined. Amplitude normalization was performed based on the waveform peak and trough value of a single cycle, and the amplitude was fully normalized as [0,1].
(3) Pulse wave single cycle length normalization: A pulse wave cycle from the pulse wave trough value to the next pulse wave trough value was defined, and the length in a single cycle to 200 points was normalized. If the length of a single cycle waveform is greater than two hundred points, the waveform in the cycle will be downsampled, otherwise the waveform in the cycle will be subjected to cubic spline interpolation.
(4) Obtaining the pulse wave template waveform: Based on average of the summation of the normalized waveform PPG_norm for the first n cycles of maintaining a constant pressure, the template formula is shown in (1):

$$PPG\_template=\frac{PPG\_norm\left(1\right)+PPG\_norm\left(2\right)+...PPG\_norm\left(n\right)}{n}$$ (1)

(5) Find the pulse wave sample waveform: Starting from the i-th (i > 2) waveform, the moving average processing is performed according to the normalized waveforms of the current n cycles, until the last waveform cycle in the data segment is added to the calculation. The calculation formula is shown in (2):

$$PPG\_sample=\frac{PPG\_norm\left(i\right)+PPG\_norm\left(i+1\right)+...PPG\_norm\left(i+n-1\right)}{n}$$ (2)

### 2.4 Feature extraction

**FIGURE 4:**
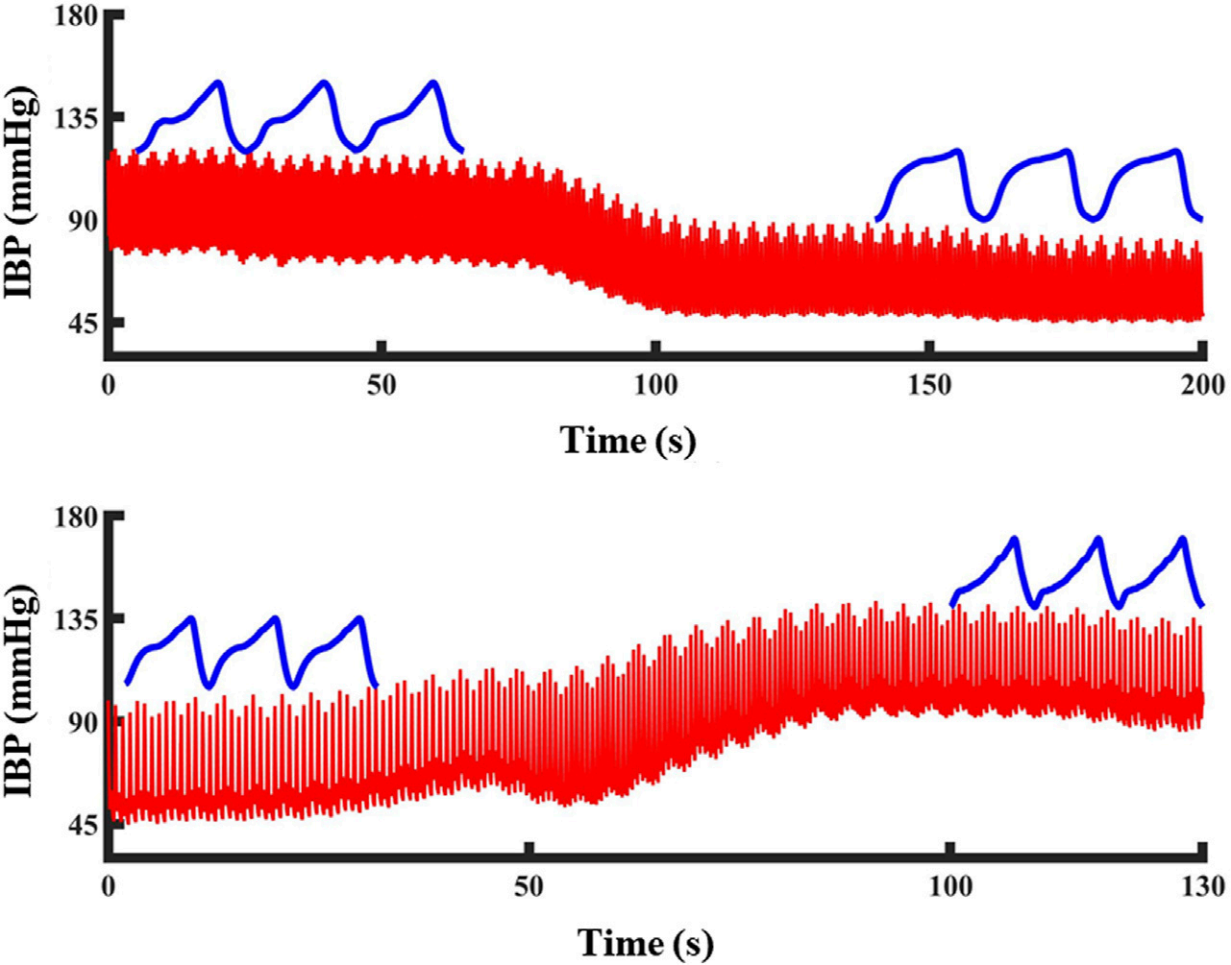
The change of PPG waveform shape during blood pressure change: Blood pressure drops and PPG waveform become shorter and wider. Blood pressure increased, PPG waveform became high and narrow. The red line represents the blood pressure and the blue line represents the PPG waveform at that blood pressure.

Three morphological features that are highly correlated with the blood pressure changes during blood loss were extracted based on previous research and the photoelectric pulse wave signal in this experiment. Figure 4 shows the changes of the pulse waveform characteristics and actual blood pressure values in the tail of the animals during blood loss and blood transfusion. In the process of blood pressure change from high to low, the shape of the pulse wave changed from normal to wider and shorter. In the process of blood pressure from small to large, the shape of the pulse wave changed from normal to thinner and taller.

**FIGURE 5:**
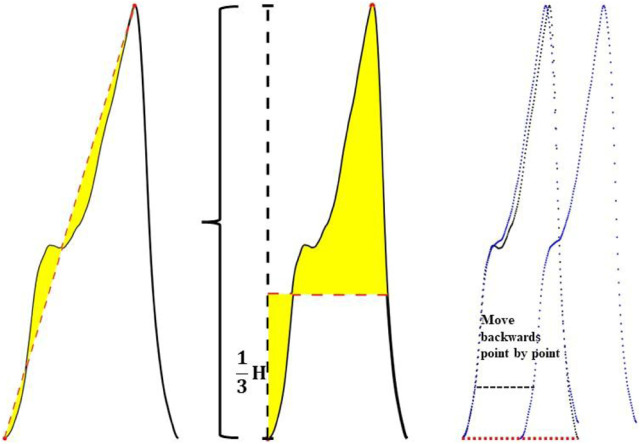
Schematic diagram of feature extraction.

According to the above pulse wave morphological changes, three morphological features in the pulse wave waveform were extracted. It includes the integral area of the rising edge of the waveform, the integral area of a single cycle of the waveform and the difference between the cross-correlation of the sample waveform and the template waveform and the autocorrelation of the template waveform. The feature extraction process is shown in Figure 5.

It includes the integral area of the rising edge of the waveform, the integral area of a single cycle of the waveform and the difference between the cross-correlation of the sample waveform and the template waveform and the autocorrelation of the template waveform.

As shown in Figure 5, the three feature extraction processes were:

Feature 1 extraction:

A linear straight line was fitted in the direction from the pulse trough value to the peak value, and the straight line was used as the baseline to obtain the integral area of the area enclosed between the rising edge curve of the waveform and the baseline.

Feature 2 extraction:

One third of the peak value of the pulse wave was taken as the baseline, and the difference between the integral area of the upper half waveform of the baseline and the lower half waveform of the baseline was calculated.

Feature 3 extraction:

Step1, The cross-correlation matrix of the sample waveform and the template waveform (R_t_-_s_) were calculated. Then the sample waveform was multiplied by the point-by-point sliding and then summed. The calculation formula is shown in (3):

$$R_{t-s}=ppg\_template\left(n\right)*{ppg\_sample}^{*}\left(-n\right)$$ (3)

Step2, The autocorrelation matrix of the template waveform (R_t_-_t_) was calculated. Autocorrelation is a special case of cross-correlation, that is, the correlation between the sequence and itself. The calculation formula is shown in (4):

$$R_{t-t}=ppg\_template\left(n\right)*{ppg\_template}^{*}\left(-n\right)$$ (4)

Step3, The difference between the cross-correlation matrix and the auto-correlation matrix was calculated, and this difference was described by the area enclosed by the two matrix curves, as shown in formula (5). When the sample waveform and the template waveform had high similarity, the area enclosed by the two matrix curves was small, otherwise, the difference between the two was considered to be greater.

$$difference=\sum_{i=1}^{n}\left|R_{t-s}\left(i\right)-R_{t-t}\;\left(i\right)\right|$$ (5)

### 2.5 Construction of blood pressure variation identification model

#### 2.5.1 Construction of blood pressure variation identification model based on single feature

Three classification models based on the classification thresholds of the three features and ten-fold cross-validation were constructed. In the process of ten-fold cross validation ten sub-data sets were randomly generated from the data set, one sub-data set was selected each time as the test set, and the ten sub-data sets were sequentially used as the test set. Using the traditional single-feature PPG single-cycle integral area as the prediction model index. The minimum and maximum sample values in the model were identified, the value was divided into 100 parts with the maximum and minimum values, and the 100 values were in turn cycled as the classification threshold. According to 10 test results (the intersection of sensitivity curve, specificity curve and accuracy curve), the optimal classification threshold of the model was selected. Finally, the test set was identified based on the optimal classification threshold, and the results were evaluated by the Accuracy (ACC) and Area Under the Curve (AUC) values.

#### 2.5.2 Construction of blood pressure variation identification model based on multiple features

**FIGURE 6:**
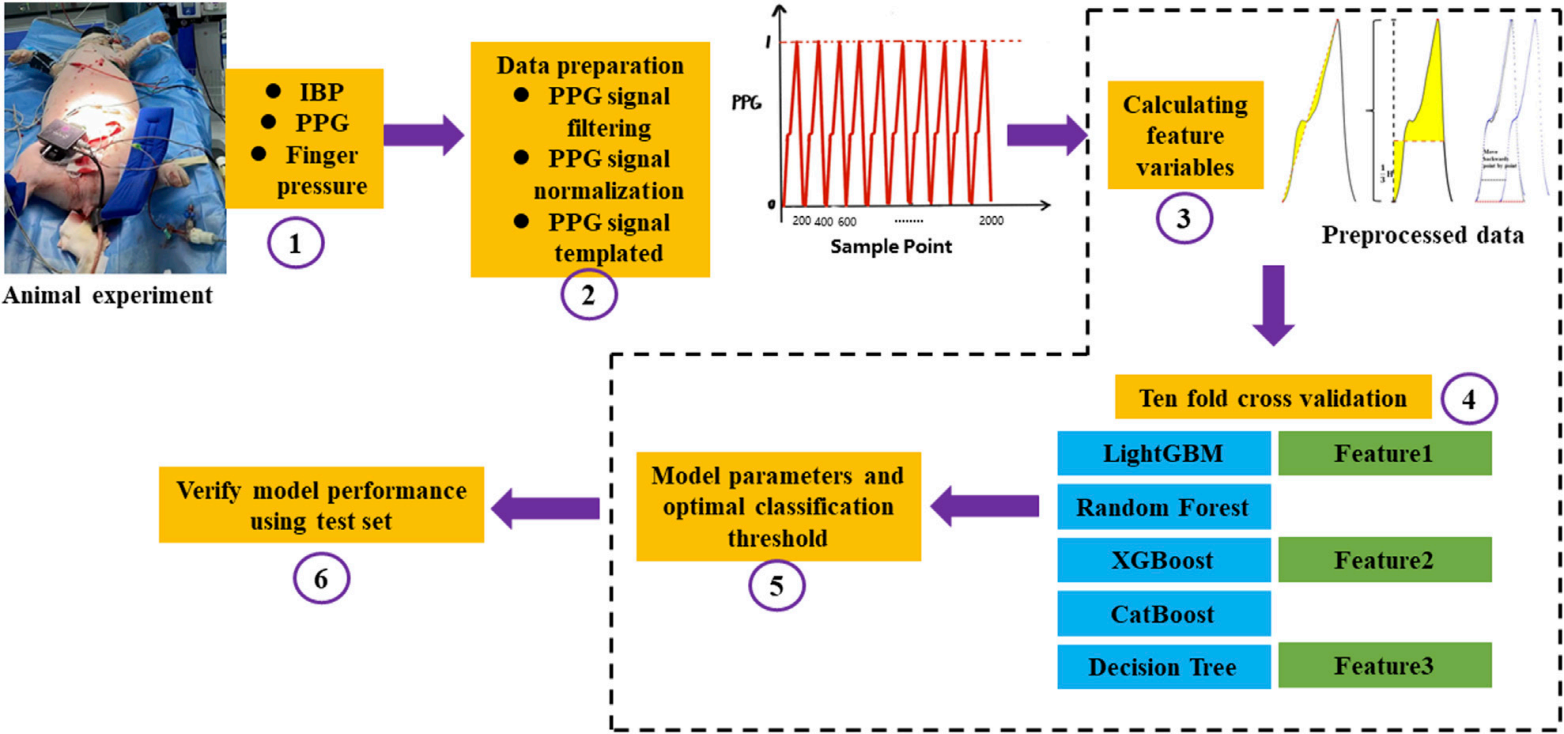
The overall flow chart of blood pressure identification. ①Firstly, the animal experiment was carried out. IBP was collected from the left femoral artery, PPG and Finger pressure were collected from the pig tail by self-developed device, and a total of three signals were collected; ②The collected PPG signals were preprocessed, the baseline drift and noise were removed by filtering, the amplitude and length of the signals were normalized, and finally the PPG signals were templated; ③Feature extraction was carried out for the preprocessed PPG signals, and the integral area of the rising edge of the waveform, the integral area of a single cycle of the waveform and the difference between the cross-correlation of the sample waveform and the template waveform and the autocorrelation of the template waveform; ④After feature extraction, the ten-fold cross-validation idea was used to construct the model. The magnitude of a single feature value is used to determine changes in blood pressure and a machine learning model with multiple features is built to identify changes in blood pressure, respectively. Feature 1 represents the integral area of the rising edge of the waveform, Feature 2 represents the integral area of the waveform in one period, and Feature 3 represents the cross correlation between the sample waveform and the template waveform and the difference between the template waveform and the template waveform; ⑤The optimal threshold points were selected by using the Youden’s index, Automatic parameter tuning using Bayesian optimization; ⑥Statistical 10-fold cross validation evaluation index.

The PPG feature sample dataset and label corresponding to the process of diastolic blood pressure, systolic blood pressure, and mean blood pressure variation of 5–15 mmHg obtained in the blood loss experiment in this study were used as input. Five classic machine learning algorithms, namely, LightGBM, Random Forest, XGBoost, CatBoost, and Decision Tree were used. The learning model performed classification and identification. The following indicators were used to evaluate the ability of five machine learning models to identify changes in blood pressure under hypovolemia. ACC represents the accuracy of the model, AUC represents the integral area under the Receiver Operating Characteristic (ROC) curve, Matthews correlation coefficient (MCC) represents the consistency of the predicted classification with the actual classification, F1 score (F1_score) considers the accuracy and recall rate of the classification model, Kappa: tests the consistency coefficient, AUPRC: the area under the precision-recall curve. Figure 6 shows the overall flow chart of blood pressure identification.

### 2.6 Constant pressure setting results

**FIGURE 7:**
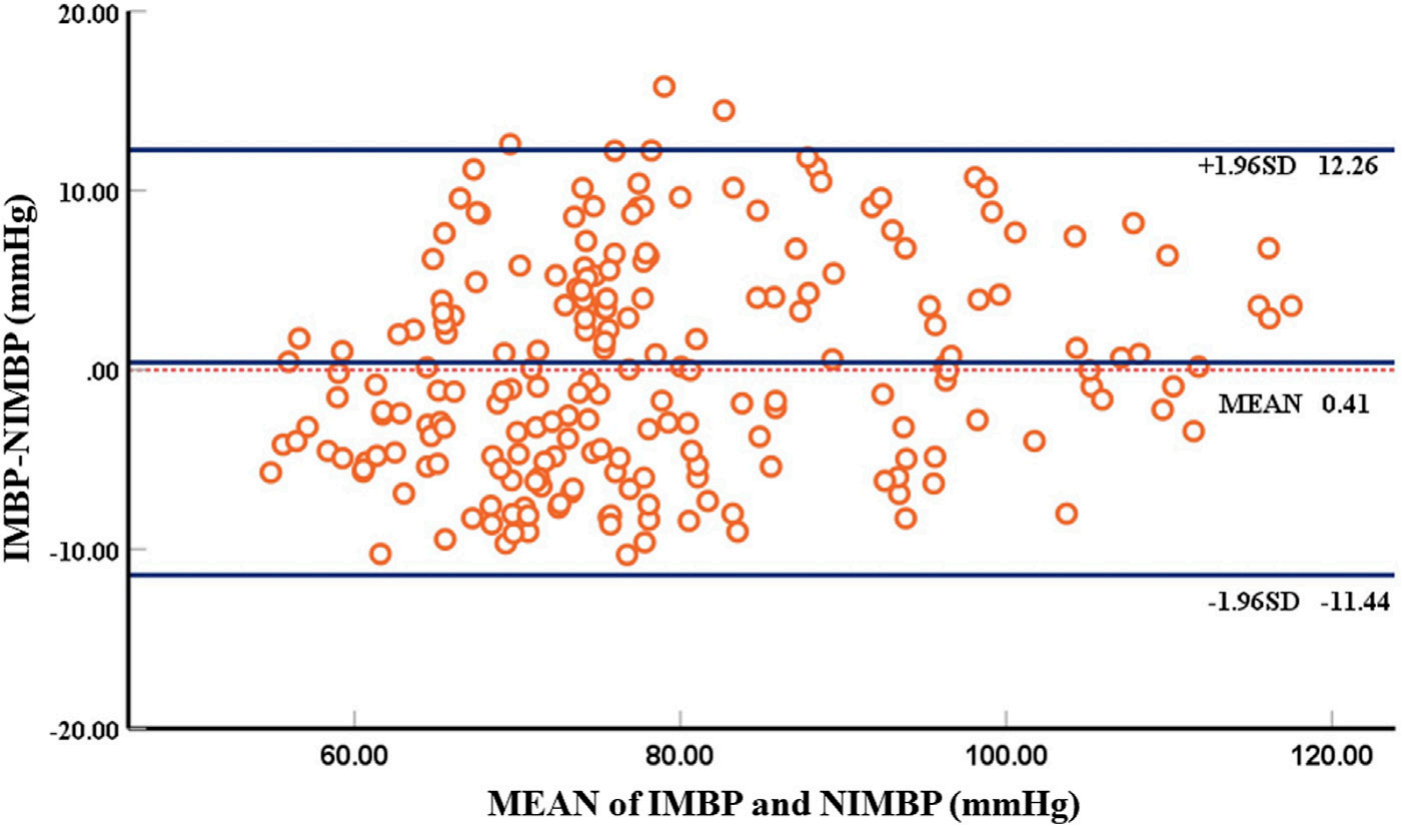
Bland-Altman plot comparing non-invasive mean blood pressure and invasive mean blood pressure. There are 214 groups of samples, with each circle representing the mean blood pressure at the beginning of identification of blood pressure.

The results of the constant pressure setting algorithm described above showed that the correlation between the non-invasive mean blood pressure of the tail and the invasive mean blood pressure of the left thigh of the animal collected simultaneously was 84%. Table 1 shows the comparison between the error analysis results of the non-invasive mean blood pressure and invasive mean blood pressure and the British Hypertension Society (BHS) standard results. The Bland-Altman analysis results are shown in Figure 7.

**TABLE 1:** Comparison with BHS standard.

Method	Subject	Cumulative error percentage (%)		
		≤5 mmHg	≤10 mmHg	≤15 mmHg
this study	NIMBP	52.8	92.5	100
BHS	Grade A	60	85	95
	Grade B	50	75	90
	Grade C	40	65	85

Using self-developed device, the mean deviation of non-invasive blood pressure detection and invasive blood pressure detection was 0.41 mmHg, and the 95% confidence of the difference between the two was −11.44 mmHg–12.26 mmHg, which can accurately detect blood pressure. According to the above Bland-Altman diagram, there are 3 samples, namely, 3/214 (1.4%), and less than 5% of the samples exceed the 95% consistency limit. The initial non-invasive mean blood pressure detection is highly consistent with the invasive mean blood pressure, which proves the reliability of the self-developed device.

### 2.7 Correlation analysis between characteristic parameters and blood pressure

**FIGURE 8:**
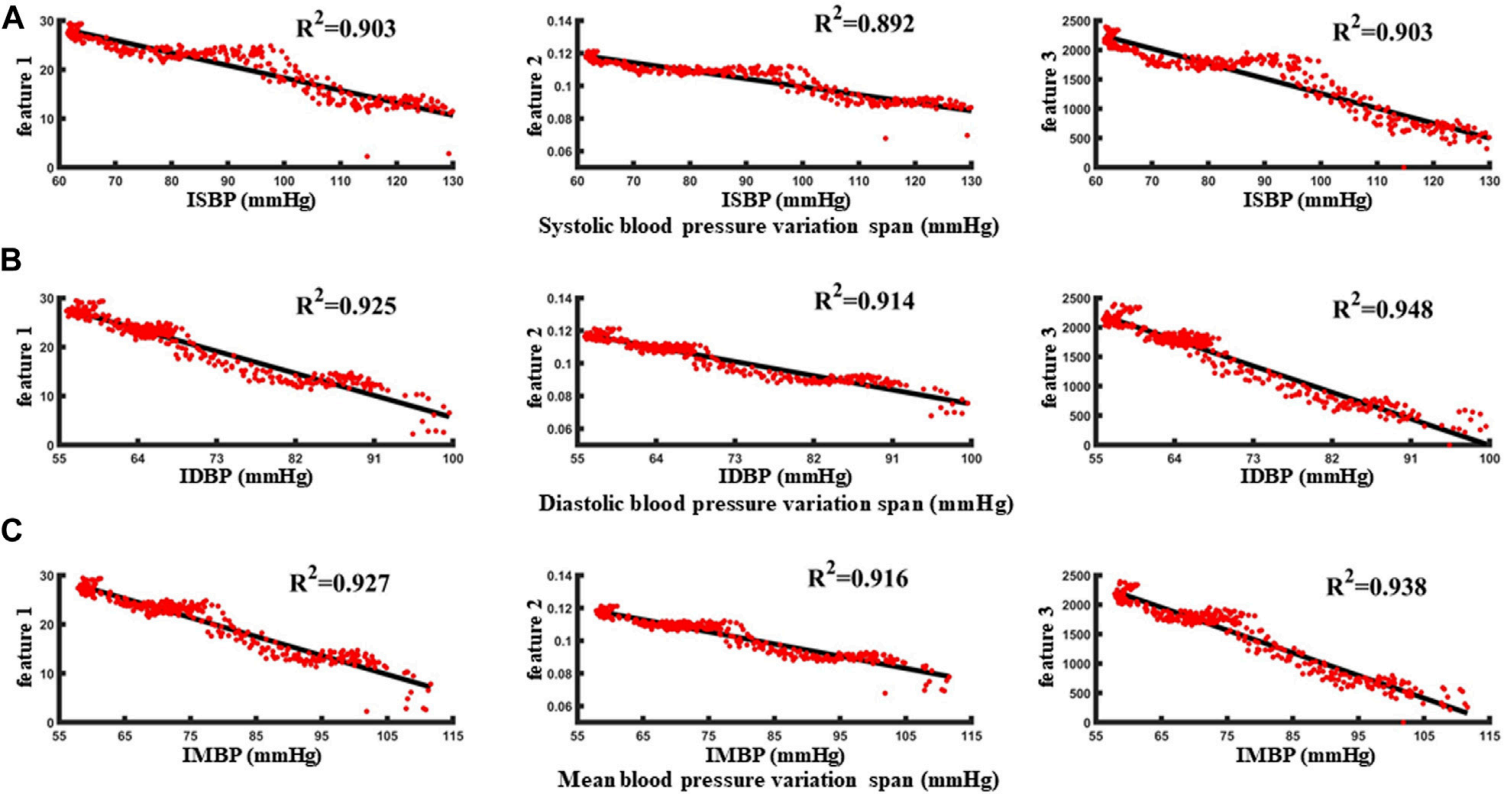
**(A–C)** are the correlation analysis of characteristic parameters with systolic blood pressure, diastolic blood pressure and mean blood pressure, respectively.

Correlation analysis was performed based on the extracted feature parameters and invasive blood pressure values to evaluate the relationship between the above three pulse wave morphological features and blood pressure. Five periodic waveform periods were used as the sliding window size to perform template processing, and the processed waveforms were the extracted features. A total of 1942 groups of valid feature samples were extracted from five animals. Three characteristic parameters and invasive blood pressure data of an animal under blood loss for 5 minutes were extracted and analyzed (Figure 8). The correlation between the two was 0.892–0.948, and the blood pressure change state under blood loss could be identified based on the characteristic parameters.

With reference to the normal fluctuation range of blood pressure within 12 h and 24 h in humans, the standard of blood pressure change was identified as 5 mmHg–15 mmHg. First, taking the blood pressure change threshold of 5 mmHg as an example, in the blood loss data segment, the samples with invasive blood pressure changes within 5 mmHg were defined as no change in blood pressure and were considered as negative sample data. Conversely, when the blood pressure was greater than 5 mmHg, the samples were identified as the occurrence of individual blood pressure during blood loss and the change was considered as positive sample data. In order to avoid over-fitting caused by the large proportion of category samples, the prediction results would be biased towards the classification with the large number of samples. The positive samples and negative samples for blood pressure change identification classification are 1:1. Taking the data set during the blood loss of a case of an animal as an example, 340 characteristic samples were extracted, and correlation analysis was carried out with the synchronously collected invasive blood pressure data (Figures 8A, B, C). The correlation between the three features and the invasive systolic blood pressure were 0.903, 0.892, and 0.903, respectively, the correlation between the three features and the invasive mean blood pressure were 0.925, 0.914, and 0.948, respectively, and the correlation between the three features and the invasive diastolic blood pressure were 0.927, 0.916, and 0.938, respectively.

### 2.8 BP variation identification model results

The data set after template processing was analyzed with five waveform periods as the sliding window size, and the blood pressure identification range was 5–15 mmHg. Variation identification accuracy and AUC values of blood pressure (diastolic blood pressure, mean blood pressure, systolic blood pressure) at 5 mmHg, 10 mmHg, 15 mmHg and average variation under 5–15 mmHg based on single-feature identification model and five machine learning models based on multi-feature are shown in Tables 2, 3, 4.

**TABLE 2:** Identification results of diastolic blood pressure variation under different models (F1 represents the classification model based on the optimal threshold of feature 1, F2 represents the classification model based on the optimal threshold of feature 2, F3 represents the classification model based on the optimal threshold of feature 3, M1 represents the LightGBM model, M2 represents the Random Forest, M3 represents XGBoost model, M4 represents CatBoost model, and M5 represents Decision Tree.).

ΔDBP (mmHg) Model	ACC (%)				AUC (%)			
	5	10	15	Mean	5	10	15	Mean
F1	79	82	90	83	80	86	90	85
F2	66	76	76	73	66	76	76	75
F3	74	80	80	78	75	81	82	79
M1	87	88	90	87	94	94	96	94
M2	83	85	93	87	95	95	97	95
M3	85	87	91	87	93	93	96	93
M4	84	87	92	88	93	95	96	94
M5	88	91	89	88	88	91	89	88

**TABLE 3:** Identification results of mean blood pressure variation under different models (F1 represents the classification model based on the optimal threshold of feature 1, F2 represents the classification model based on the optimal threshold of feature 2, F3 represents the classification model based on the optimal threshold of feature 3, M1 represents the LightGBM model, M2 represents the Random Forest, M3 represents XGBoost model, M4 represents CatBoost model, and M5 represents Decision Tree).

ΔMBP (mmHg) Model	ACC (%)				AUC (%)			
	5	10	15	Mean	5	10	15	Mean
F1	82	85	93	86	82	87	93	88
F2	65	75	77	73	67	77	79	75
F3	83	86	90	86	85	84	90	87
M1	78	89	94	89	92	96	98	96
M2	82	91	95	90	92	96	98	96
M3	79	90	94	89	92	96	97	95
M4	77	90	94	89	92	96	98	96
M5	83	91	93	90	83	91	93	90

**TABLE 4:** Identification results of systolic blood pressure variation under different models (F1 represents the classification model based on the optimal threshold of feature 1, F2 represents the classification model based on the optimal threshold of feature 2, F3 represents the classification model based on the optimal threshold of feature 3, M1 represents the LightGBM model, M2 represents the Random Forest, M3 represents XGBoost model, M4 represents CatBoost model, and M5 represents Decision Tree.).

ΔSBP (mmHg) Model	ACC (%)				AUC (%)			
	5	10	15	Mean	5	10	15	Mean
F1	82	83	86	84	84	84	88	85
F2	64	74	77	73	65	76	79	74
F3	78	82	85	83	80	83	85	84
M1	83	90	91	90	93	96	96	95
M2	80	90	93	89	94	97	96	96
M3	84	90	93	90	92	96	96	95
M4	82	91	90	89	93	96	97	96
M5	86	91	89	90	86	91	89	90

As shown in Tables 2, 3, 4: the blood pressure variation identification model under hypovolemia in this study was constructed based on the two newly extracted feature parameters, compared with the traditional Feature 2. The reported ACC and AUC values for diastolic blood pressure increased with the ∆DBP more than 5%, while the ACC and AUC values for mean blood pressure and systolic blood pressure increased with the ∆MBP and ∆SBP more than 10%. The identification model results based on multi-dimensional features compared with the traditional feature 2 blood pressure variation identification model showed that the diastolic blood pressure variation identification ACC and AUC increased with the ∆DBP 14%–15% and 15%–20%, respectively; the mean blood pressure variation average identification ACC and AUC were increased with the ∆MBP 16%–17% and 15%–21%, respectively, and the mean identification of systolic blood pressure variation ACC and AUC were increased with the ∆SBP 16%–17% and 16%–22%, respectively. Comparison of the results of multi-dimensional-based blood pressure variation identification under hypovolemia showed that the features proposed in this study are better for mean blood pressure and systolic blood pressure variation identification than diastolic blood pressure fluctuation identification under hypovolemia. Furthermore, the analysis of the results of the five machine learning models showed that the variation identification accuracy of the mean blood pressure and systolic blood pressure in the range of 5–15 mmHg under the random forest and decision tree machine learning models can reach 90% and all the AUC values exceeding 90%.

## 3 Analysis and discussion

### 3.1 Comparative analysis of different blood pressure identification range results

**FIGURE 9:**
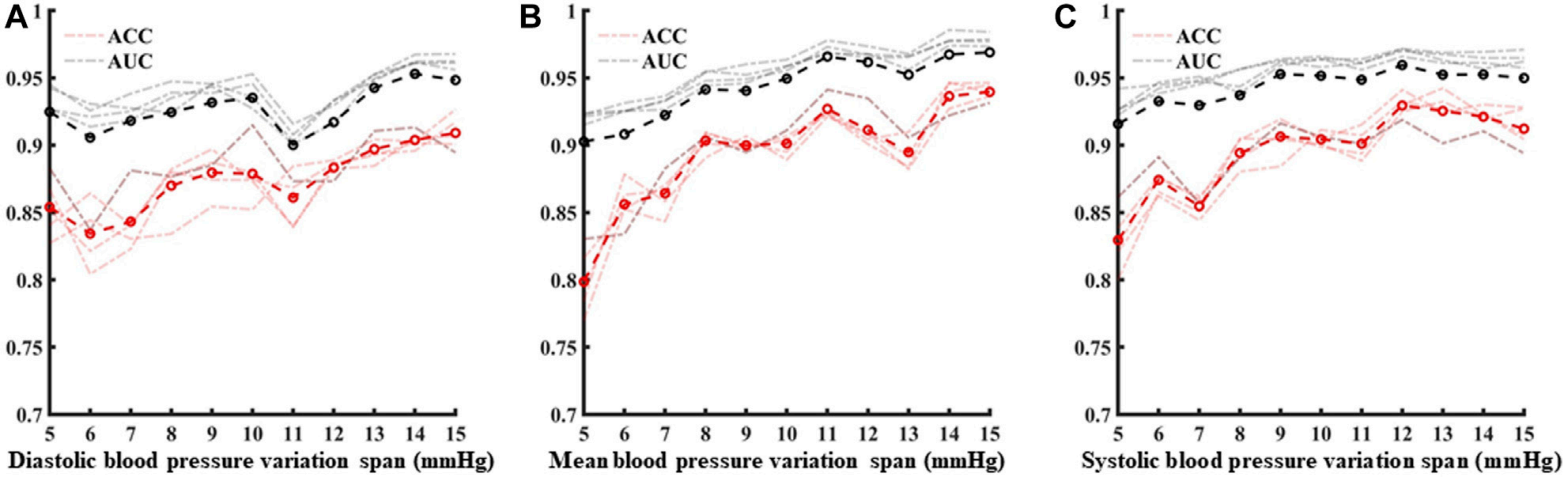
**(A–C)** are the analysis of identification results under different blood pressure variability under diastolic blood pressure, mean blood pressure and systolic blood pressure, respectively.

Following template processing, the data set was analyzed with five periodic waveforms as the sliding window size, and blood pressure variation identification was performed for 5 mmHg–15 mmHg in turn. Based on three characteristics of systolic blood pressure, diastolic blood pressure, and average blood pressure variation identification model using the five machine learning models, the average ACC and the average AUC were calculated (Figure 9). With the increase of blood pressure variation range, the ACC values and AUC values show an overall increasing trend.

### 3.2 Comparison of identification results of blood pressure changes under different template waveforms

**FIGURE 10:**
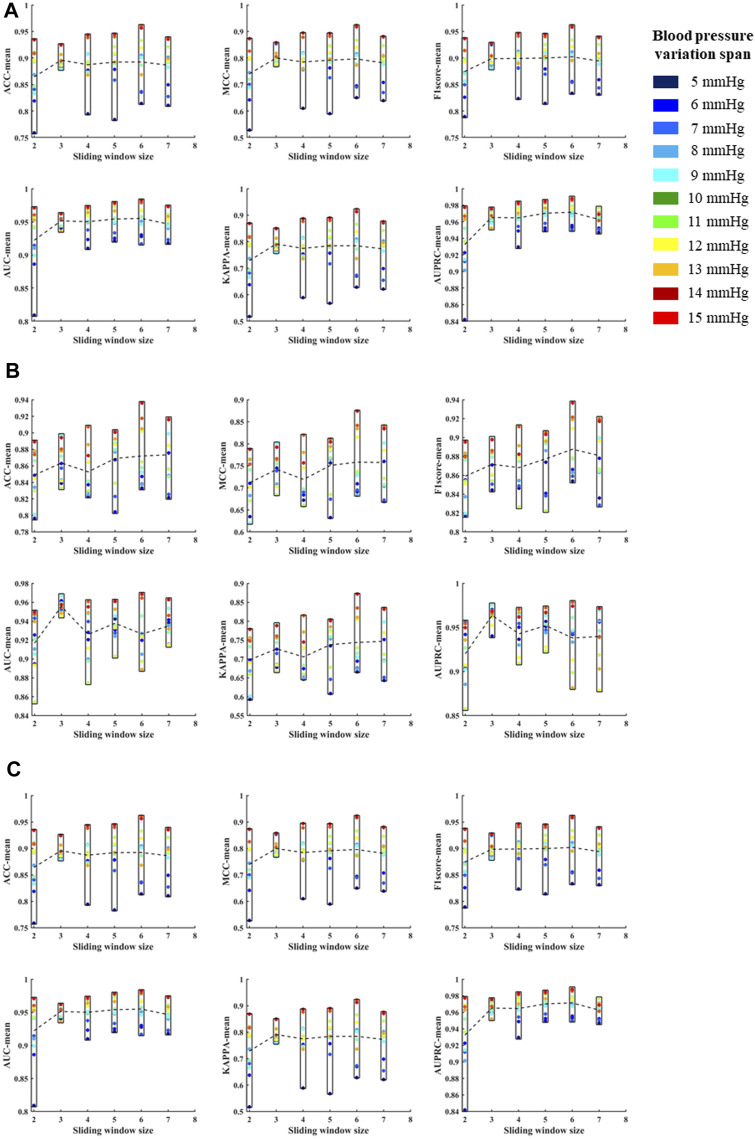
**(A)** is the mean values of ACC, MCC, F1 score, AUC, KAPPA and AUPRC for systolic blood pressure at different sliding window sizes, in models established for blood pressure changes spanning 5–15 mmHg, respectively. **(B)** is the mean values of ACC, MCC, F1 score, AUC, KAPPA and AUPRC for diastolic blood pressure at different sliding window sizes, in models established for blood pressure changes spanning 5–15 mmHg, respectively. **(C)** is the mean values of ACC, MCC, F1 score, AUC, KAPPA and AUPRC for mean blood pressure at different sliding window sizes, in models established for blood pressure changes spanning 5–15 mmHg, respectively. The X label of each graph represents the size of the sliding window and the Y label represents the different evaluation metrics for blood pressure changes across 5–15 mmHg under the current sliding window.

**FIGURE 11:**
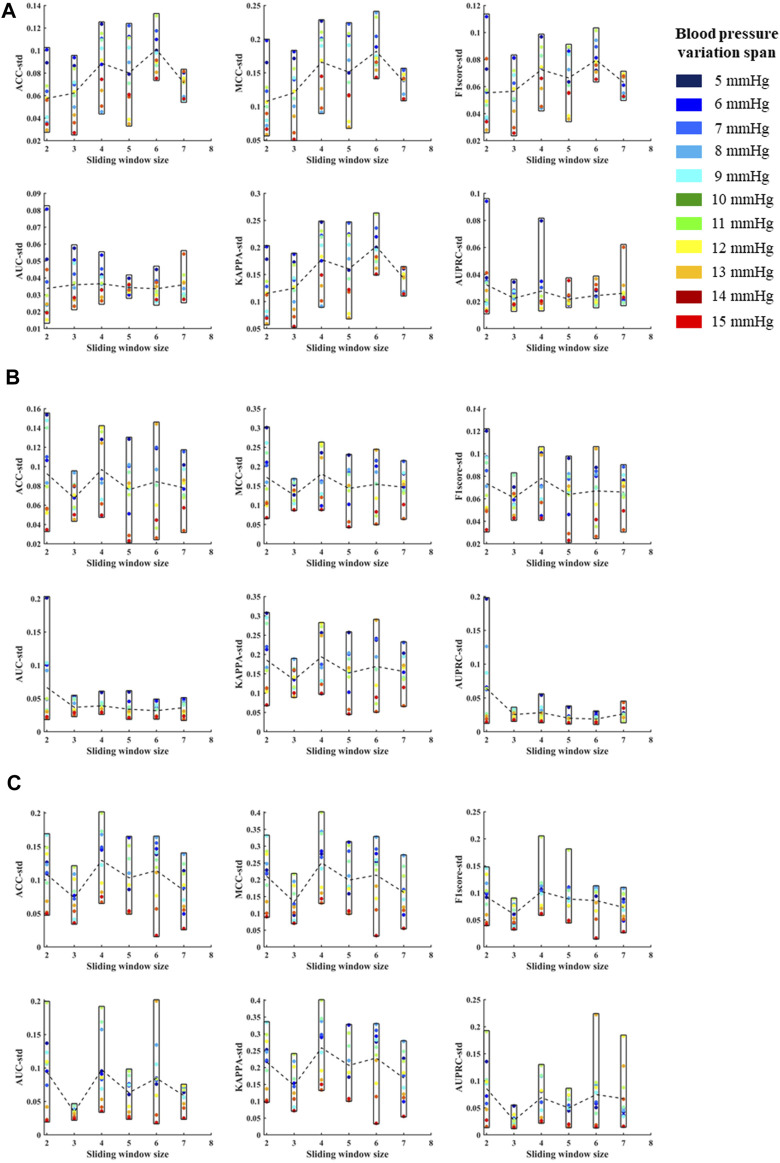
**(A)** is the standard deviations of ACC, MCC, F1 score, AUC, KAPPA and AUPRC for systolic blood pressure at different sliding window sizes, in models established for blood pressure changes spanning 5–15 mmHg, respectively. **(B)** is the standard deviations of ACC, MCC, F1 score, AUC, KAPPA and AUPRC for diastolic blood pressure at different sliding window sizes, in models established for blood pressure changes spanning 5–15 mmHg, respectively. **(C)** is the standard deviations of ACC, MCC, F1 score, AUC, KAPPA and AUPRC for mean blood pressure at different sliding window sizes, in models established for blood pressure changes spanning 5–15 mmHg, respectively. The X label of each graph represents the size of the sliding window and the Y label represents the different evaluation metrics for blood pressure changes across 5–15 mmHg under the current sliding window.

According to the above template processing process, five cycles of pulse waves were selected for sliding average processing. In order to compare the optimal sliding window size, this study repeats the above feature extraction process and modeling process for waveforms processed based on two to seven cycles of pulse waves. Based on different template waveforms, blood pressure changes were identified for 5–15 mmHg in turn, and the mean and standard deviation of the systolic blood pressure, diastolic blood pressure, and mean blood pressure variation identification indicators under the above-mentioned multi-feature identification model were used to analyze the mean value and standard deviation, respectively (Figures 10, 11). The results in the two figures show that the average value and standard deviation of the indicators of different template waveforms are comprehensively compared, and the blood pressure change identification effect is the best based when the three-cycle pulse wave was used as the template-processed waveform of the sliding window size.

### 3.3 Limitation

In this work, we used 1942 samples of 5 pigs to get the result, in the early stage to verify the feasibility of our method, and later experiments with larger samples data will be done to further improve its stability and extensibility.

## 4 Conclusion

New features and the blood pressure variation identification models under hypovolemia are proposed and established in this study, based on the morphological characteristics of photoplethysmography wave in the tail of animals. The results showed that the morphological characteristic parameters of the volumetric pulse wave under constant pressure can effectively and accurately identify the degree of blood pressure variation under blood loss. Compared with the traditional features, the two new features can further improve the accuracy of the traditional volumetric compensation method to capture blood pressure variation under low perfusion. Compared with single feature models, the classification model based on multi-dimensional features can achieve better identification effect. The feature proposed in this study is more suitable for the variation identification of mean blood pressure and systolic blood pressure, compared with the fluctuation identification of diastolic blood pressure under low blood volume. The results of blood pressure identification at different levels of 5–15 mmHg proposed in this paper can provide information of blood pressure variation for patients with mild blood loss or hemorrhagic shock, and provide non-invasive continuous blood pressure change warning for different clinical application scenarios. Furthermore, the new morphological features proposed in this study can provide an additional new blood pressure tracking method for the continuous non-invasive blood pressure monitoring equipment based on the volume compensation method.

## Data Availability

The raw data supporting the conclusion of this article will be made available by the authors, without undue reservation.
